# Plasma microRNA-145-5p as a diagnostic biomarker for acute deep vein thrombosis

**DOI:** 10.1016/j.rpth.2024.102671

**Published:** 2024-12-31

**Authors:** Christopher Antoun, Vårin Eiriksdatter Wikan, Øyvind Øverli, Thor Ueland, Gholamreza Jafari Yeganeh, Sigrid Kufaas Brækkan, Ellen Brodin, John-Bjarne Hansen

**Affiliations:** 1 Thrombosis Research Group, Department of Clinical Medicine, UiT–The Arctic University of Norway, Tromsø, Norway; 2 Division of Internal Medicine, Department of Haematology, Akershus University Hospital, Lørenskog, Norway; 3 Division of Internal Medicine, University Hospital of North Norway, Tromsø, Norway; 4 Research Institute of Internal Medicine, Oslo University Hospital, Rikshospitalet, Oslo, Norway; 5 Faculty of Medicine, Institute of Clinical Medicine, University of Oslo, Oslo, Norway; 6 Department of Emergency Medicine, Akershus University Hospital, Lørenskog, Norway

**Keywords:** biomarker, cross-sectional studies, deep vein thrombosis, diagnosis, microRNA, patients

## Abstract

**Background:**

Identification of new biomarkers for acute deep vein thrombosis (DVT) that could lower the need for diagnostic imaging to confirm or rule out the diagnosis would be advantageous. microRNA-145-5p (miR-145) has the potential to be a diagnostic marker for acute DVT, but its diagnostic performance has not been evaluated in consecutive patients referred to the hospital with suspected DVT.

**Objectives:**

The aim of this study was to assess the diagnostic performance of miR-145 for the diagnosis of acute lower extremity DVT.

**Methods:**

Patients consecutively referred to the emergency room at Akershus University Hospital (Norway) due to suspicion of acute DVT between June 2021 and July 2023 were included. Within 24 hours of admission, blood was collected for assessment of plasma miR-145 (index test), and whole-leg compression ultrasound (reference standard) was used to confirm or rule out DVT. Cohen’s d effect size and area under the receiver operating curve (AUC) were used to estimate the diagnostic performance of miR-145 and D-dimer.

**Results:**

Among 360 included patients, 101 (28%) were diagnosed with DVT. miR-145 showed poor diagnostic performance, as indicated by a Cohen’s d of −0.002 (95% CI, −0.241 to 0.216) and an AUC of 0.59 (95% CI, 0.51-0.67). For D-dimer, Cohen’s d was 1.66 (95% CI, 1.43-1.89), and the AUC was 0.92 (95% CI, 0.86-0.96).

**Conclusion:**

Plasma levels of miR-145 showed poor diagnostic performance for lower extremity acute DVT among persons referred to the emergency room with clinical signs and symptoms of DVT.

## Essentials

• Effective diagnosis of DVT remains a clinical challenge.
• The diagnostic potential of plasma miR-145 was evaluated in consecutive patients with suspected DVT.
• miR-145 did not discriminate DVT patients from those without DVT with similar signs and symptoms.
• There is still a lack in diagnostic biomarkers to reduce reliance on resource-intensive imaging.

## Graphical abstract

**Figure:**
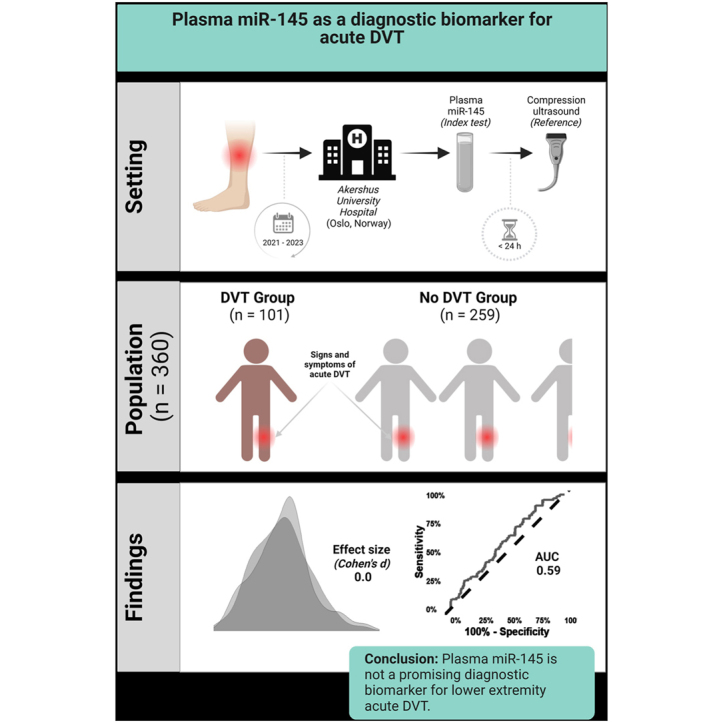

## 1 Introduction

Acute deep vein thrombosis (DVT) can be challenging to diagnose due to unspecific symptoms and signs [1]. For patients referred to the emergency room with a suspected DVT, a clinical algorithm using pretest probability assessment and D-dimer is currently recommended to identify those in whom DVT can be immediately ruled out and those who require further diagnostic imaging to confirm or rule out the diagnosis [2]. Even with good adherence to this diagnostic algorithm, only 20% to 35% of those referred to imaging have a DVT diagnosis [3]. Excess use of diagnostic imaging results in increased use of time, resources, and costs for both patients and healthcare systems [4]. Therefore, it would be desirable to identify new biomarkers for acute DVT that could improve the diagnostic algorithm and thereby lower the proportion of patients in whom imaging would be required to confirm or rule out the diagnosis.

MicroRNAs (miR) are short, single-stranded RNAs that downregulate gene expression and have emerged as promising biomarker candidates owing to their presence and stability in plasma [5,6]. Sahu et al. [7] reported lower levels of plasma miR 145-5p (miR-145) in acute venous thromboembolism (VTE) patients (*n* = 20) than in controls (*n* = 20) and that miR-145 could discriminate between VTE patients and controls with an area under the receiver operating curve (AUC) of 0.95. The authors additionally reported that the administration of miR-145 mimics lowered thrombosis formation in a rat stasis model [7]. Subsequently, Morelli et al. [8] showed in a population-based case-cohort of 510 VTE cases and 1890 subcohort participants that high plasma levels of miR-145 were associated with a 48% decreased risk of future VTE.

Although the findings of plasma miR-145 as a potential diagnostic biomarker for VTE are promising, validation in a larger study that reflects the regular clinical setting is required. To the best of our knowledge, no previous study has examined the diagnostic performance of miR-145 among unselected patients referred to the emergency room with suspected DVT. Therefore, the aim of our study was to investigate the diagnostic performance of miR-145 for assessment of acute lower extremity DVT with compression ultrasound (CUS) as the reference standard and compare it with the diagnostic performance of D-dimer in a cross-sectional study of patients consecutively referred to the hospital emergency room with suspected DVT.

## 2 Methods

### 2.1 Study design and population

We performed a prospective diagnostic cross-sectional study of patients referred to the emergency department at Akershus University Hospital (Ahus) in Oslo, Norway, for suspicion of acute DVT. The study was approved by the Regional Committee for Health and Research Ethics (approval number: 200878), and informed written consent was obtained in accordance with the Declaration of Helsinki. The patients were enrolled consecutively between June 2021 and July 2023 following referral by either their primary healthcare provider or an emergency physician. Patients presenting with symptoms of lower extremity DVT (ie, swelling, redness, venous ectasia, and/or pain) who were aged 18 to 80 years, used Ahus as their local hospital, and provided consent to participate were considered eligible for inclusion. There were no restrictions with regard to pretest probability or D-dimer results before inclusion. Inpatients were not eligible for this study. Further exclusion criteria were previous history of VTE, life expectancy <2 years, ongoing therapeutic anticoagulant treatment (except for those receiving a single dose of anticoagulation due to DVT suspicion), and if the reference standard (ie, CUS) or index test assessment (ie, miR-145 levels) was not performed or had inconclusive results.

For the index test, blood was collected from an antecubital vein into vacutainer tubes containing citrate using a 21-gauge needle. Blood was centrifuged at 2500 × *g* for 15 minutes at room temperature, and the remaining platelet-poor plasma was aliquoted and stored at −80 °C. The blood sample was either taken at the same time as the routine blood work was performed or separately after the reference standard test, both performed within 24 hours of admission. All patients were subjected to whole-leg CUS (ie, reference standard) performed by radiologists or certified emergency physicians who were blinded to the result of the index test. A noncompressible proximal (at the site of or proximal to *vena poplitea*) or distal (distal to *vena poplitea*) deep vein was the diagnostic criterion for the presence of DVT, while patients with compressible deep veins were classified as no-DVT. Whole-leg CUS is acknowledged as an objective diagnostic tool for DVT, with established sensitivity and specificity of 94% and 97%, respectively [9]. Computed tomography venography was not used as a reference standard to facilitate a direct comparison of the index test to the diagnostic performance of CUS. Patients with DVT and concurrent pulmonary embolism (PE) or superficial vein thrombosis were included, whereas patients with isolated PE were excluded. Furthermore, patients with DVT requiring catheter-directed thrombolysis were excluded, while patients with isolated superficial vein thrombosis were classified as no-DVT. Intramuscular vein thrombosis was classified as DVT. All no-DVT patients were followed for 3 months after the inclusion date to assess the rate of CUS misclassification for either DVT or PE.

Information on patient characteristics, DVT characteristics (description and duration of symptoms and localization of thrombus), comorbidities, differential diagnoses in no-DVT patients, as well as results from standard admission blood work (including D-dimer) and initiation of anticoagulation pending diagnostic confirmation with CUS, were retrieved by review of medical records. D-dimer was measured by standard immunoturbidimetric methods on a Roche Cobas t711 using the Tina-quant D-Dimer Gen.2 assay (D-DI2; Roche Diagnostics).

### 2.2 Measurement of plasma miR-145 levels

Plasma samples were shipped on dry ice to Qiagen Genomic Services for miR quantification with quantitative polymerase chain reaction (qPCR) analysis. The samples were thawed on ice and subsequently centrifuged at 3000 × *g* for 5 minutes at 4 °C. Total RNA was extracted from each sample using the miRNeasy Plasma Advanced Kit (Qiagen) [10], and the RNA was eluted in a final volume of 50 μL using RNase-free water. For quantification of miR-145, 2 μL of RNA were reverse transcribed into complementary DNA (cDNA) using the miRCURY LNA RT Kit (Qiagen) [11]. This cDNA was then diluted 50-fold and subjected to polymerase chain reactions (PCR) according to the miRCURY LNA miRNA PCR protocol [12]. A single qPCR assay for miR-145 was conducted using a specialized miRNA PCR panel, which included the SYBR Green master mix (Qiagen). This panel also contained reference genes, controls for RNA purification and cDNA synthesis verification, markers for contamination, a standard for plate-to-plate comparison, and a blank control without any sample. PCR amplification was performed in a Roche LightCycler 480 system, accommodating 384 samples per run. The PCR results, including quantification cycle values and melting curves, were analyzed using Roche’s software.

The efficiency of RNA isolation and cDNA synthesis was assessed using spike-ins. Consistency across different PCR plates was ensured by evaluating the interplate calibrator, and hemolysis was assessed by considering 2 miR ratios. Uniform results for these quality checks indicated that RNA extraction, cDNA synthesis, and qPCR were performed successfully. Samples affected by hemolysis and those who failed the quality checks were removed from the study. The raw quantification cycle values of miR-145 were normalized by the expression of miR-425-5p, as this miR has been previously used as a reference in several miR studies and is stably expressed in plasma. We confirmed this choice using both the geNorm [13] and BestKeeper [14] algorithms. Of note, the personnel involved in the quantification of miR-145 in plasma were blinded to the result of the CUS (ie, DVT status) and to any clinical information of the patients.

### 2.3 Statistical analysis

The required sample size was based on an effect size for miR-145 that was large enough to be considered clinically significant. Clinical significance was defined to be the minimum effect size that would allow a 10% decrease in the mean proportion of CUS referrals when DVT is suspected. The expected proportions of CUS were derived from previous reports [3,[15], [16], [17]] and modeled into a normal distribution. Our estimated minimum effect size translated into a Cohen’s d of 0.57, which was smaller than the derived Cohen’s d effect size of approximately 2.04 observed in a previous miR-145 study [7], thus allowing considerable adjustment for publication bias and inflated diagnostic measures. Assuming a DVT prevalence of 30% and striving for a study power of 95% with a 5% significance level, at least 66 participants would be required in the DVT group and 165 in the no-DVT group. To accommodate a potential 30% loss due to withdrawal of consent or poor sample quality, we set out to recruit 94 participants in the DVT group and 235 in the no-DVT group.

Characteristics of patients with and without DVT were described as proportions for categorical variables and as median with IQR for continuous variables. To compare the plasma levels of miR-145 between the 2 groups, we calculated the Cohen’s d effect size, with corresponding 95% CIs through bootstrapping with 5000 permutations, as implemented in the DABEST package [18]. Typically, a Cohen’s d effect size of 0.2 would be interpreted as small, 0.5 as medium, and 0.8 as large [19]. Because sex differences in plasma miR-145 levels have been previously reported [20], we calculated the Cohen’s d effect size for males and females separately. For comparison, we also calculated the Cohen’s d for D-dimer. Receiver operating characteristic curves derived from logistic regression models were used to evaluate the diagnostic performance of plasma miR-145 and D-dimer for the diagnosis of acute DVT, and the AUC was estimated. All statistical analyses were conducted using R version 4.3.2 (R Core Team, 2023).

## 3 Results

**Figure 1:**
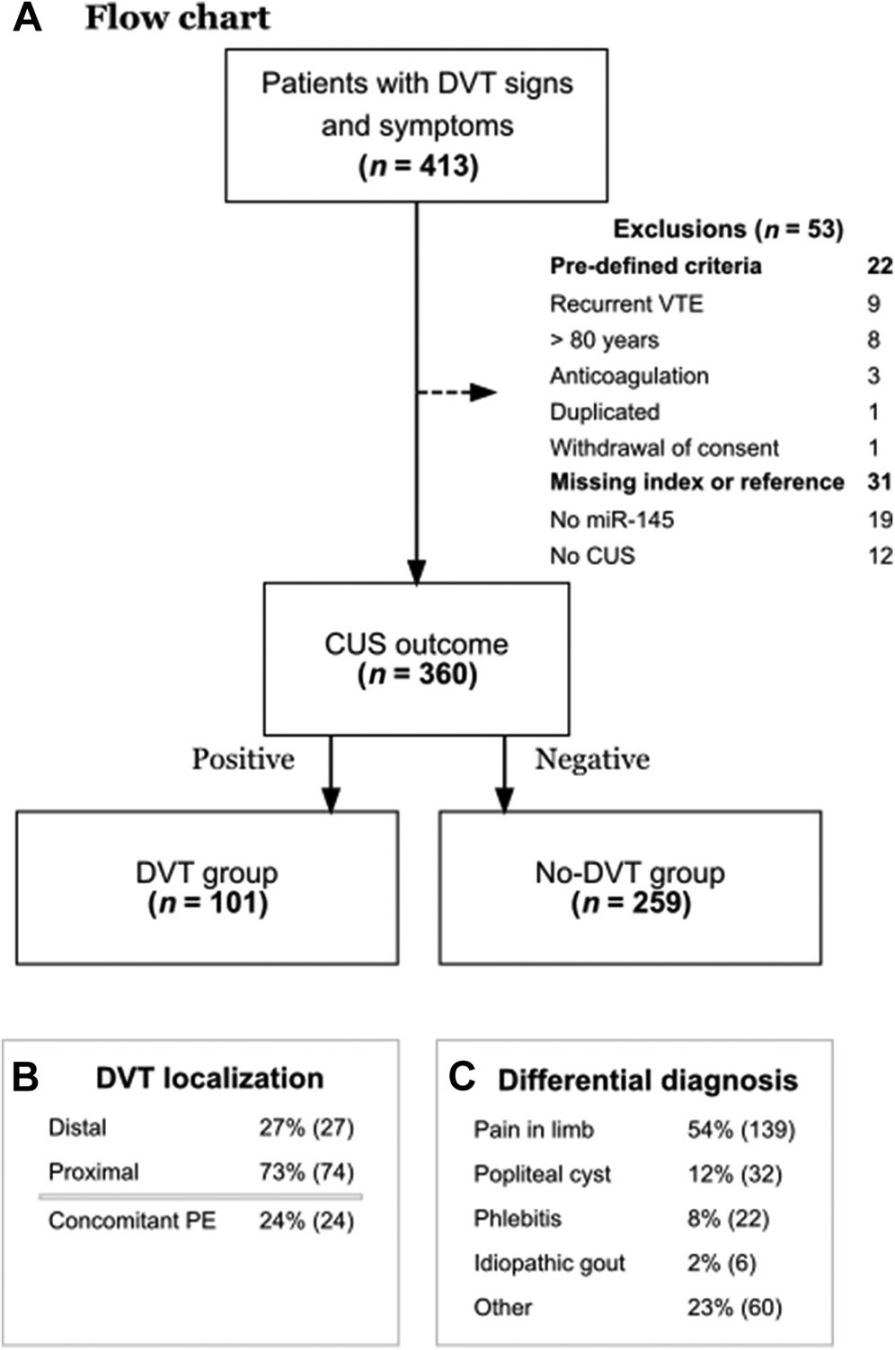
Overview of study participant flow. (A) Participant flowchart with exclusions based on predefined criteria and missing index or reference standards. (B) Localization of deep vein thrombosis (DVT) for the DVT group. (C) Differential diagnosis for the no-DVT group. CUS, compression ultrasound; miR-145, microRNA-145-5p; PE, pulmonary embolism; VTE, venous thromboembolism.

A total of 413 patients with symptoms and signs of acute DVT in their lower extremities were considered for inclusion, of which 360 were included after the application of the inclusion and exclusion criteria (Figure 1). Of these, 101 (28%) had a confirmed DVT diagnosis, while the diagnosis was ruled out in 259 patients (Figure 1A). Among the confirmed DVTs, 73% were located in proximal veins and 27% in distal veins (Figure 1B). Additionally, 26% of the cases had a concomitant PE, and among these, 4% were associated with distal DVT. For patients in whom DVT was ruled out, the most common differential diagnoses were pain in the limb (54%), popliteal cyst (12%), phlebitis (superficial vein thrombosis; 8%), and idiopathic gout (2%; Figure 1C, Supplementary Table). The misclassification rate of CUS for DVT was about 1%. Among patients in the no-DVT group, 3 were later diagnosed with DVT and 1 with PE within 3 months of follow-up. No patient experienced any adverse effects in relation to performing the index test or the reference standard.

### 3.1 Baseline characteristics of DVT and no-DVT groups

Characteristics of the study population stratified by DVT status are presented in Table 1. The median age (58 years) was similar in the 2 groups. Notably, the proportion of females was lower in the DVT (37%) group than in those without DVT (60%), while the proportion of overweight individuals (38% vs 26%) and patients who had undergone surgery (17% vs 11%) was higher in those with DVT than in those without. A higher proportion of the DVT patients had received a single dose of anticoagulation before the CUS examination (45% vs 35%). As expected, median D-dimer (3.4 vs 0.5 mg/L) and C-reactive protein levels (17 vs 3 mg/L) were higher and showed higher IQR variability in the DVT group than in the no-DVT group.

**Table 1:** Distribution of baseline characteristics for the study population and for the deep vein thrombosis and no deep vein thrombosis groups.

Characteristics	DVT (*n* = 101)	No-DVT (*n* = 259)
Age, y	58 (67-48)	58 (49-70)
Female sex	37 (37)	60 (155)
Cancer	8 (8)	9 (23)
CVD	5 (5)	7 (18)
Overweight	38 (38)	26 (68)
Pregnancy or postpartum	1 (1)	2 (4)
Single-dose anticoagulation	45 (45)	35 (90)
Surgery or trauma	17 (17)	11 (28)
Symptom duration (d)	4 (3-4)	4 (3-4)
CRP (mg/L)	17.0 (5.9-40.0)	3.0 (1.3-9.2)
D-dimer (mg/L)	3.4 (1.8-6.9)	0.5 (0.3-0.9)

Values are presented as % (*n*) for categorical variables and median (IQR) for continuous variables. CVD includes previous myocardial infarction, stroke, and/or heart failure. The overweight classification was based on a report from a physical examination or a body mass index > 25 kg/m^2^. Surgery or trauma indicates whether the patient underwent surgery or had physical trauma within the past 3 months. A single-dose anticoagulant was prescribed due to the current suspicion of DVT.

CRP, C-reactive protein; CVD, cardiovascular disease; DVT, deep vein thrombosis.

### 3.2 Diagnostic performance of miR-145 and D-dimer

**Figure 2:**
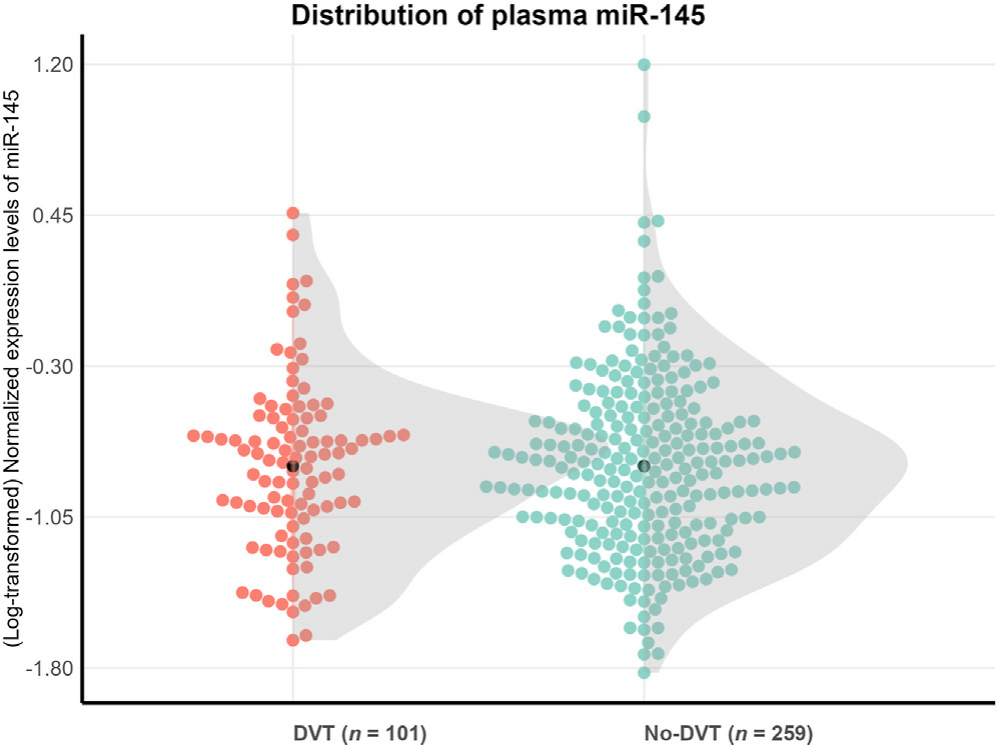
Distribution of plasma microRNA-145-5p (miR-145) levels in the deep vein thrombosis (DVT) and no-DVT groups. Colored points represent individual observations. The shaded areas depict the overall distribution trends, while the black dots mark the mean values for each group.

**Figure 3:**
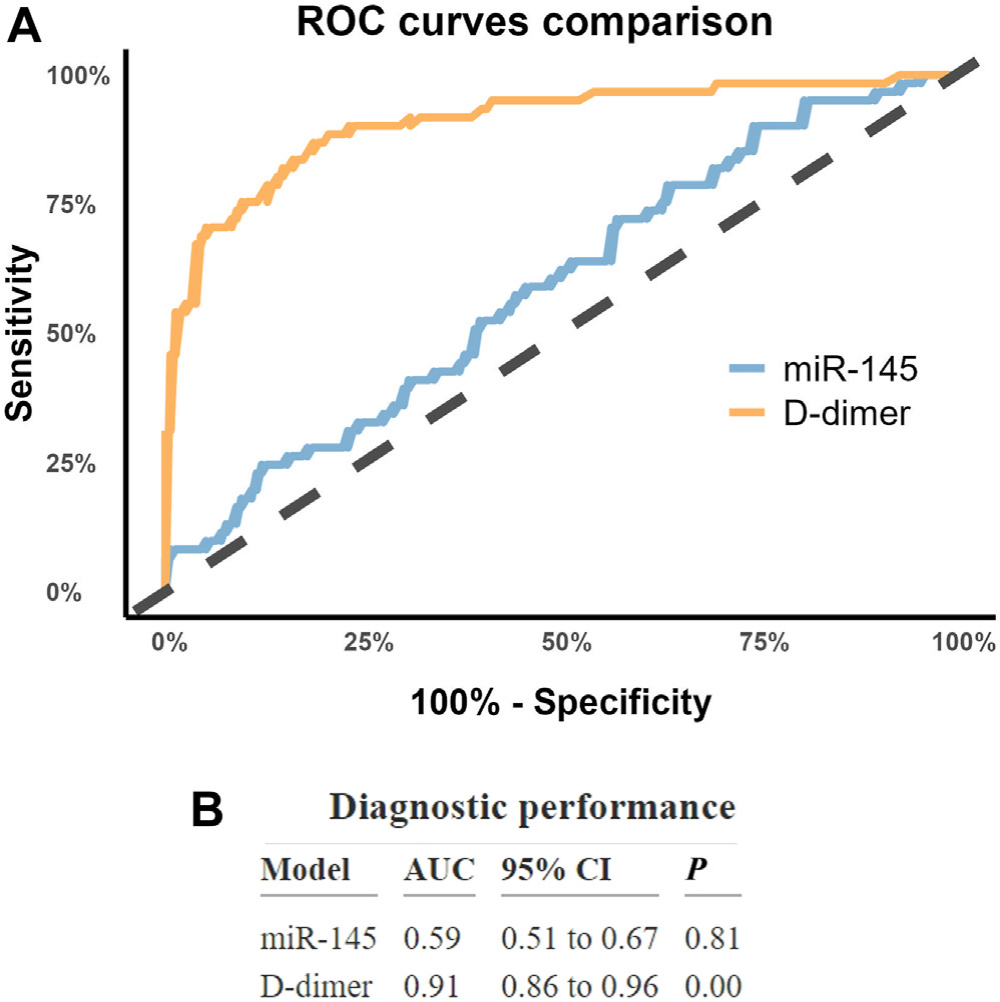
Diagnostic performance of microRNA-145-5p (miR-145) and D-dimer. (A) Receiver operating curves (ROC) comparing miR-145 (blue) and D-dimer (orange). The dashed black line represents the line of no discrimination. (B) Area under the receiver operating curves (AUC) with 95% confidence interval (CI) from the logistic regression model.

The mean value (−0.796 vs −0.797) and distributions of log-transformed miR-145 levels did not differ between the DVT group and the no-DVT group (Figure 2). The derived Cohen’s d effect size of miR-145 was −0.002 (95% CI, −0.24 to 0.22; Table 2). When stratified by sex, the Cohen’s d for miR-145 was 0.30 (95% CI, −0.02 to 0.63) for males and −0.05 (95% CI, −0.39 to 0.29) for females (Table 2). The Cohen’s d effect size for D-dimer was 1.66 (95% CI, 1.43-1.89) for the total study population (Table 2). The receiver operating characteristic curves for miR-145 and D-dimer are shown in Figure 3A. For miR-145, the AUC was 0.59 (95% CI, 0.51-0.67), and the AUC for D-dimer was 0.92 (95% CI, 0.86-0.96; Figure 3B).

**Table 2:** Cohen’s d effect sizes with 95% CIs for between-group comparison (deep vein thrombosis and no deep vein thrombosis) of microRNA-145-5p (overall and sex-stratified) and D-dimer.

Test	Cohen’s d	95% CI
**miR-145**		
Overall	−0.002	−0.24 to 0.22
Female	−0.05	−0.39 to 0.29
Male	0.30	−0.02 to 0.63
**D-dimer**		
Overall	1.66	1.43-1.89

miR-145, microRNA-145-5p.

## 4 Discussion

Our study evaluated plasma miR-145 as a diagnostic biomarker for acute DVT among patients referred to the emergency room with signs and symptoms of DVT. We found that miR-145 had a poor diagnostic performance for acute DVT with a Cohen’s d effect size of −0.002 and an AUC of 0.59. For comparison, the Cohen’s d of D-dimer was 1.66, while the AUC was 0.92. Our findings suggest that plasma miR-145 is not useful as a diagnostic biomarker for acute DVT.

The results of our study are in contrast to the previous findings of Sahu et al. [7], who reported an AUC for plasma miR-145 of 0.95 for VTE diagnosis in a small study of 20 VTE patients and 20 controls. This discrepancy in results can be attributed to major differences between the studies with regard to study design, study size, and study populations. Notably, the cases in the study by Sahu et al. [7] were young (median age, 31.5 years), exclusively male, and recruited among military servants, while our study included men and women recruited from a general population with a median age (58 years) in line with that reported in other unselected DVT populations [3,21,22]. Furthermore, the study by Sahu et al. [7] included a high proportion of VTEs at rare sites (20% of the cases had cerebral sinuous thrombosis, and 10% had portal vein thrombosis), while 55% had lower extremity DVT and 15% of patients had PE. In addition, patients with any systemic comorbidities, cancer, vasculitis, or prior surgery were excluded. There was limited information about the selection process and clinical characteristics of the controls and whether they displayed any symptoms of VTE [7]. The inclusion of highly selected VTE patients and comparison with healthy controls without any other diseases would likely overestimate the diagnostic performance of a biomarker [23], and results may not be generalizable to a more diverse population with clinical symptoms of DVT and comorbidities. Furthermore, the small study size could make the study more vulnerable to chance findings. In contrast, our cross-sectional diagnostic study was substantially larger than the study by Sahu et al. [7], and it was designed to closely mimic the clinical setting by consecutive enrollment of adult persons of both sexes with signs and symptoms indicative of lower extremity DVT. We had few exclusion criteria, and as a result, common differential diagnoses of DVT were included in the no-DVT group. Thus, our findings indicated that in the regular clinical setting, miR-145 could not be used as a diagnostic biomarker for acute DVT.

Given that sex differences in the levels of miR-145 have been observed [20], we analyzed the diagnostic performance of miR-145 separately for males and females. Although the effect size was somewhat larger in males than in females, a Cohen’s d of 0.3 indicated a poor diagnostic utility of miR-145 in males.

While plasma miR-145 did not meet the criteria for being a diagnostic biomarker [24], a few previous studies have indicated a role of miR-145 in the pathogenesis of VTE [8]. In a nested case-control study, we found that miR-145 levels were inversely associated with future risk of VTE in the general population [8]. Moreover, Sahu et al. [7] showed that intervention with miR-145 mimics was associated with decreasing thrombus size in a mouse stasis model of VTE. Notably, a good diagnostic biomarker is often a result of a specific pathophysiological response (ie, consequence) of a disease and is not necessarily involved in the pathogenesis [24]. Thus, the poor diagnostic utility of miR-145 for acute DVT observed in the present study does not preclude a potential role of miR-145 in the pathogenesis of DVT.

Strengths of our study include the cross-sectional design with consecutive enrollment of persons referred to diagnostic work-up with signs and symptoms of lower extremity DVT, the thorough timing and assessment of index (miR-145) and reference tests (CUS), as well as blinding of staff who performed CUS with regards to an individual’s miR-145 levels (and *vice versa*, those who assessed miR levels were blinded to the CUS result), which are all in line with the guidelines given by the standards for reporting diagnostic accuracy studies [25]. The 3-month follow-up of patients without DVT enabled the calculation of misclassification rates and minimized the risk of bias from an imperfect gold standard. Furthermore, our study had sufficient statistical power to detect any meaningful effect of miR-145. Using Cohen’s d as the measure for standardized effect size, our sample size was large enough to detect a “medium” effect if it existed. Some limitations merit attention. Similar to previous diagnostic studies on VTE [21], the prevalence of DVT in our study population was slightly higher than that observed in routine clinical practice (28% vs 15%-20%) [3,15]. According to an overview of systematic reviews, one of the drivers for patients to participate in a study is personal benefit, which includes closer monitoring and gaining knowledge about one’s own health [26]. This might translate to a slightly higher inclination of patients diagnosed with DVT to participate in our study, leading to the observed prevalence of DVT in our sample. A higher prevalence could potentially overestimate the diagnostic performance [27], but as we found a poor diagnostic performance of miR-145, this would not affect the overall conclusions of our study. Moreover, the results of our study predominantly apply to people of Caucasian descent.

In conclusion, plasma levels of miR-145 showed poor diagnostic performance for lower extremity acute DVT among persons referred to the emergency room with clinical signs and symptoms of DVT. The diagnostic utility of miR-145 was substantially weaker than for the established biomarker D-dimer.
