# Supplementary material for: Plasma microRNA-145-5p as a diagnostic biomarker for acute deep vein thrombosis
Source: Res Pract Thromb Haemost. 2024 Dec 31;9(1):102671. doi: 10.1016/j.rpth.2024.102671 (PMC11788850; doi:10.1016/j.rpth.2024.102671)
Supplement: Supplementary Table [file mmc1.docx]

**Supplementary Table.** Distribution of all given differential diagnosis (n = 259) based on the international classification of diseases version 10 (ICD-10) for the no-DVT patients. The column “Category” indicates the higher-level categorization of each ICD-10 used to present differential diagnosis in this study.

| **Differential diagnosis** | **ICD-10** | **Category** | **n** |
| --- | --- | --- | --- |
| Achilles tendinitis | M76.6 | Other | 3 |
| Acute subendocardial myocardial infarction type 1 | I21.41 | Other | 1 |
| Allergic urticaria | L50.0 | Other | 1 |
| Aneurysm and dissection of artery of lower extremity | I72.4 | Other | 1 |
| Arthritis, unspecified; calf/knee | M13.96 | Other | 1 |
| Benign lipomatous neoplasm of skin and subcutaneous tissue of limbs | D17.2 | Other | 1 |
| Calcaneal spur | M77.3 | Other | 2 |
| Cellulitis, unspecified | L03.9 | Other | 3 |
| Contusion of knee | S80.0 | Other | 1 |
| Contusion of other and unspecified parts of lower leg | S80.1 | Other | 1 |
| Dyspnea | R06.0 | Other | 1 |
| Erysipelas | A46 | Other | 2 |
| Idiopathic gout | M10.0 | Idiopathic gout | 6 |
| Idiopathic gout; ankle/foot/toe | M10.07 | Other | 1 |
| Intermittent hydrarthrosis; calf/knee | M12.46 | Other | 1 |
| Intra-abdominal lymph nodes | C77.2 | Other | 1 |
| Localized oedema | R60.0 | Other | 5 |
| Localized swelling, mass and lump, lower limb | R22.4 | Other | 4 |
| Localized swelling, mass and lump, unspecified | R22.9 | Other | 1 |
| Lyme disease | A69.2 | Other | 1 |
| Lymphoedema, not elsewhere classified | I89.0 | Other | 1 |
| Muscle strain | M62.6 | Other | 1 |
| Myalgia; calf/knee | M79.16 | Other | 2 |
| Oedema, unspecified | R60.9 | Other | 1 |
| Other meniscus derangements | M23.2 | Other | 1 |
| Other specific arthropathies, not elsewhere classified | M12.8 | Other | 1 |
| Other specified hemorrhagic conditions | D69.8 | Other | 1 |
| Pain in joint | M25.5 | Other | 2 |
| Pain in joint; calf/knee | M25.56 | Other | 2 |
| Pain in limb | M79.6 | Pain in limb | 8 |
| Pain in limb; ankle/foot/toe | M25.57 | Pain in limb | 1 |
| Pain in limb; calf/knee | M79.66 | Pain in limb | 129 |
| Pain in limb; pelvis/thigh | M79.65 | Pain in limb | 1 |
| Peroneal tendinitis | M76.7 | Other | 1 |
| Phlebitis and thrombophlebitis of other deep vessels of lower extremities | I80.2 | Phlebitis | 8 |
| Phlebitis and thrombophlebitis of other sites | I80.8 | Phlebitis | 7 |
| Phlebitis and thrombophlebitis of superficial vessels of lower extremities | I80.0 | Phlebitis | 6 |
| Phlebitis and thrombophlebitis of unspecified site | I80.9 | Phlebitis | 1 |
| Residual foreign body in soft tissue; pelvis/thigh | M79.55 | Other | 1 |
| Rupture of popliteal cyst | M66.0 | Popliteal cyst | 32 |
| Secondary malignant neoplasm of bone and bone marrow | C79.5 | Other | 1 |
| Soft tissue disorder, unspecified | M79.9 | Other | 1 |
| Spontaneous ecchymoses | R23.3 | Other | 1 |
| Synovial cyst of popliteal space [Baker] | M71.2 | Other | 4 |
| Varicose veins of lower extremities without ulcer or inflammation | I83.9 | Other | 6 |
